# Factors Associated with Retention of HIV Patients on Antiretroviral Therapy in Care: Evidence from Outpatient Clinics in Two Provinces of the Democratic Republic of the Congo (DRC)

**DOI:** 10.3390/tropicalmed7090229

**Published:** 2022-09-05

**Authors:** Gulzar H. Shah, Gina D. Etheredge, Lievain Maluentesa Nkuta, Kristie C. Waterfield, Osaremhen Ikhile, John Ditekemena, Bossiky Ngoy Belly Bernard

**Affiliations:** 1 Jiann-Ping Hsu College of Public Health, Georgia Southern University, Statesboro, GA 30460, USA; 2 FHI 360, Washington, DC 20009, USA; 3 FHI 360, Kinshasa 1015, Congo; 4 National Multisectoral HIV/AIDS Program (PNMLS), HIV Program, Presidency of DRC, Kinshasa 1013, Congo

**Keywords:** HIV, retention, antiretroviral therapy, Democratic Republic of Congo, health disparities, loss to follow-up, WHO clinical stage

## Abstract

Interruptions in the continuum of care for HIV can inadvertently increase a patient’s risk of poor health outcomes such as uncontrolled viral load and a greater likelihood of developing drug resistance. Retention of people living with HIV (PLHIV) in care and determinants of attrition, such as adherence to treatment, are among the most critical links strengthening the continuum of care, reducing the risk of treatment failure, and assuring viral load suppression. Objective: To analyze the variation in, and factors associated with, retention of patients enrolled in HIV services at outpatient clinics in the provinces of Kinshasa and Haut-Katanga, Democratic Republic of the Congo (DRC). Methods: Data for the last visit of 51,286 patients enrolled in Centers for Disease Control (CDC)-supported outpatient HIV clinics in 18 health zones in Haut-Katanga and Kinshasa, DRC were extracted in June 2020. Chi-square tests and multivariable logistic regressions were performed. Results: The results showed a retention rate of 78.2%. Most patients were classified to be at WHO clinical stage 1 (42.1%), the asymptomatic stage, and only 3.2% were at stage 4, the severest stage of AIDS. Odds of retention were significantly higher for patients at WHO clinical stage 1 compared to stage 4 (adjusted odds ratio (AOR), 1.325; confidence interval (CI), 1.13–1.55), women as opposed to men (AOR, 2.00; CI, 1.63–2.44), and women who were not pregnant (vs. pregnant women) at the start of antiretroviral therapy (ART) (AOR, 2.80; CI, 2.04–3.85). Odds of retention were significantly lower for patients who received a one-month supply rather than multiple months (AOR, 0.22; CI, 0.20–0.23), and for patients in urban health zones (AOR, 0.75; CI, 0.59–0.94) rather than rural. Compared to patients 55 years of age or older, the odds of retention were significantly lower for patients younger than 15 (AOR, 0.35; CI, 0.30–0.42), and those aged 15 and <55 (AOR, 0.75; CI, 0.68–0.82). Conclusions: Significant variations exist in the retention of patients in HIV care by patient characteristics. There is evidence of strong associations of many patient characteristics with retention in care, including clinical, demographic, and other contextual variables that may be beneficial for improvements in HIV services in DRC.

## 1. Introduction

Regardless of the WHO clinical stage, interruptions in the continuum of HIV care can inadvertently increase a patient’s risk of poor health outcomes such as uncontrolled viral load [1], drug resistance [2,3], increased HIV transmission [3,4], ineffective treatment [2], and mortality [3]. These potential consequences are especially detrimental to children because the disease progresses more rapidly in them compared to adults [5]. In the Democratic Republic of the Congo (DRC), the HIV incidence rate per 1000 population for all ages was recently reported as 0.18 [0.12–0.28]. Until now, there has been limited research into how retention rates (also known as “continuity of treatment”) affect people living with HIV [5]. The continuum of care for successful HIV treatment includes HIV testing, linkage to care, retention in care, and viral load testing and suppression [6]. Retention in care is a critical component of a successful treatment; people living with HIV (PLHIV) who are enrolled in antiretroviral therapy (ART) programs routinely receive services befitting their clinical needs and have the best outcomes [7,8,9]. In general, PLHIV are considered lost to follow up (LTFU) if there is no recorded visit within 180 days of their previous visit, or after they are expected for an appointment [10]. Nonetheless, many countries in the last few years have began shortening that LTFU time frame. In the DRC, it was only recently updated to 90 days after the last appointment as a matter of national policy. Failing to adhere to HIV treatment and thus being labeled as LTFU could be due to discontinued ART, self-transfer to another program, or death; regardless of the reason, LTFU causes the patient to be included in the clinic’s patient attrition rate [4].

Many factors may be considered indicators for HIV patient retention and attrition at treatment centers in countries such as the DRC, such as varying treatment protocols for how patients initiate treatment, poor quality of care, transportation, or a caregiver’s HIV status [9]. In Sub-Saharan recent studies found that 40% of PLHIV were classified as LTFU [4]. LTFU rates among children are high, especially among infants whose mothers had yet to initiate ART at the time they were enrolled [9,11,12]. It varied from 62 to 95% depending on the length in the program; in the DRC, the retention rate was approximately 79.1% [9].

A study conducted in Kinshasa showed that pregnant women at care enrollment were more likely not to return, however, among those who attended at least one follow-up visit, there were no statistically significant differences in loss to care by pregnancy status [13]. These studies also found that retention rates were significantly higher in HIV-specialized facilities like health, education, action, leadership (HEAL) Africa and Amo-Congo/Kasa-Vubu compared to general care facilities [9]. Patients with low viral failure rates [14] shared their HIV status [15], and those who lived in rural communities had a strong community support organization [9]. Better health information systems to allow patients to be tracked between service delivery points are needed to properly evaluate pre-ART loss to care [16,17]. Overarching strategies to increase retention are those that address the individual patient needs well as the general needs of the larger population. To initiate ART earlier [8,16,18] cash transfers [19,20] are suggested in the literature.

There are gaps in the literature regarding how demographic and clinical characteristics affect retention and attrition of PLHIV in resource-limited countries, such as the DRC. It is unclear how factors such as rurality and urbanicity, duration on ART, multi-month dependence on ART, and advanced WHO stage at initiation could affect retention and attrition rates. The objective of this study was to examine these factors as they apply to HIV-infected people in the Democratic Republic of the Congo.

## 2. Methods

### 2.1. Study Design and Data

#### 2.1.1. Data

Data for this retrospective cohort study were extracted in June 2020 from 241 CDC-funded HIV/AIDS outpatient clinics in two DRC provinces: Haut-Katanga and Kinshasa. Clinics that did not participate in the CDC-funded program were excluded. The study data pertained to all PLHIV who were on ART and had at least one clinic visit to any of the HIV clinics between January 2017 and April 2020. 

Although the outcome variable (retention vs. attrition) was collected based on the date of the most recent visit, the most recent visit of some of the PLHIVs predated the October 2017 implementation of the updated national policy that allowed patients to begin ART on the same day they received a positive HIV test [21]. Data security was maintained, and patient confidentiality was assured. All data were secondary and no identifying information was included in the data obtained for this study. The original data were collected under a local IRB approval No. ESP/CE/229/2019 by FHI 360. Georgia Southern University (protocol number HI 9260) exempted this research from a full IRB review.

#### 2.1.2. Population and Setting

The study setting was the DRC provinces Haut-Katanga and Kinshasa. Haut-Katanga is a region known for its large copper and cobalt mines and has a population of approximately 5.7 million; Kinshasa is the capital city and has a population of approximately 14.5 million [22]. The health zones contributing data included Binza Ozone, Kambove, Kikula, Kilela Balanda, Kimbanseke, Kingabwa, Kinshasa, Likasi, Limete, Lingwala, Lukafu, Masina I, Matete, Mont-Nafula1, Mufunga Sampwe, Ndjili, Ngaba, and Nsele. Four of these 18 health zones are classified by the DRC general secretary of health as rural, and the other 14 as urban. These clinics are owned by the DRC government or faith-based organizations or are private facilities where HIV/AIDS activities were integrated by the National HIV/AIDS Program (PNLS) with the support of the President’s Emergency Plan for AIDS Relief (PEPFAR) through CDC implementing partners. These partners—ICAP, Humana People to People (HPP), and Sante Rurale (SANRU)—share the data from this research. The clinics conduct HIV counseling, testing and treatment as well as service delivery data using an Electronic Patient Management System (EMS) called Tier.Net (TIER.Net Version 1.10, Cape Town University, Cape Town, South Africa) has been used.

### 2.2. Measures

#### 2.2.1. Dependent Variables

Variable retention was put into operation if a patient on ART had not had a lapse of 180 days or longer since the last clinic visit or expected date of return. The original variable had four attributes for each of the visits as recorded by the clinical staff at Tier.net: (a) died, (b) on ART, (c) transferred out, and (d) lost to follow-up (LTFU). The variable retention was coded “1” if the patient met the retention definition and was recorded as “on ART” or coded “0” if the patient met the definition of attrition and coded as “died” or “LTFU.” Patients who were transferred to another facility (3% of all cases) were not included in this analysis.

#### 2.2.2. Independent Variables 

The demographic independent variables, sex and age, were collected at the time of enrollment in the facility and modified as needed (e.g., age). The age at last visit was coded as younger than 15, 15 to <55 years, and 55 years or older; age was calculated by the patient’s date of birth and the date of their last visit. The contextual and clinical variables included pregnancy status at ART initiation and coded as yes, no, not reported, or not applicable (i.e., the patient was male or <15 years); the WHO clinical stages from 1 (asymptomatic) to 4 (the severest stage of AIDS) [23], or stage unknown; whether multiple months of ART were provided at the last visit and coded as a one-month supply, two or more months’, or none; and duration under ART in months coded as <7, 7 to <22, 22 to <53, and ≥53 months based on quartiles as cut points. Duration was calculated as the time between ART initiation and the date of the last visit. The clinical facility location variables were coded as urban or rural based on the health zone, rurality/urbanicity classification, and the province of the clinic’s location—Haut-Katanga, or Kinshasa.

#### 2.2.3. Analytical Methods

We computed descriptive statistics such as frequency distribution, and the percentages for all independent and dependent variables. To describe the level of variation in dependent variable retention by various demographic, clinical, and facility characteristics, we used Chi-square. To assess the association of each independent variable with the dependent variable (retention) after statistically controlling the effect of covariates, we computed multivariable logistic regression. Measures of predictive power such as Cox and Snell R Square and Nagelkerke R Square and Hosmer–Lemeshow goodness-of-fit tests were used to determine the model’s fitness. All analyses for this study were performed using IBM SPSS Statistics version 25.0 (IBM Corporation, Armonk, NY, USA) [24].

## 3. Results

A total of 51,286 patients were included in the analysis. A retention rate of 78.2% was found for PLHIV receiving ART at HIV/AIDS clinics of the National HIV/AIDS Program (PNLS) in Kinshasa and Haut-Katanga provinces, whereas the attrition rate was 21.8% (Table 1). A large majority, 78.4%, were aged 15 to <55, and patients younger than 15 constituted 7.1%, while those aged 55 or older accounted for 14.5%. The distribution of patients according to the WHO’s four hierarchical clinical stages was 42.1% at stage 1 (asymptomatic), 18.7% at stage 2, 22.6% at stage 3, and 3.2% at stage 4 (the severest stage). Roughly a quarter of the patients had been on ART for 53 months or longer, whereas 23.2% had been on ART at the last visit for less than 7 months. Roughly one in two (48.2%) patients had multiple months of supply at the last visit, whereas 44.8% received one-month ART; the remaining 7% received no ART. Descriptive statistics for other variables are presented in Table 1.

### 3.1. Variation in Retention

The variation in the percentage of patients retained was significant at *p* < 0.001 for all clinical and demographic characteristics included in the Chi-square analysis (Table 2). Retention rates in care were the lowest for patients in later WHO clinical stages, 72.4% for stage 3, and 67.0% for stage 4 compared to 79.7% for stage 1 and 79.4% for stage 2. The percentages of patients retained in care by the number of months for which ART was dispensed indicated that those receiving multiple months of supply had the highest chance of retention (84.8%). In contrast, those who did not receive any ART at the last visit had the lowest retention (17.6%), and those receiving a one-month supply showed a moderate chance of retention, 67.3%. Duration on ART was also associated with variation in retention, with the highest chance of retention, 84.5%, for patients in the 2nd quartile (i.e., duration on ART was 7 to <23 months), and the lowest retention (71.7%) for those who had been on ART for 53 months or longer. Women who were pregnant at the ART initiation had slightly lower retention rates than those who were not pregnant, 87.0% and 88.6%, respectively.

Retention rates were the lowest for patients younger than 15 and the highest for those aged 55 or older (73.2 vs. 80.7%; *p* < 0.001). Females had lower chances of retention than males: 77.4 and 79.9%, respectively. Clinics in rural health zones had higher retention, 92.5%, compared to urban clinics, 77.7%. Retention rates were higher for the clinics in Haut-Katanga province than in Kinshasa (93.7 vs. 76.6%).

### 3.2. Logistic Regression of Factors Affecting Odds of Retention

The fitness of the logistic regression model was shown by the Hosmer–Lemeshow goodness-of-fit test, which resulted in a Chi-square of 107.5; *p* < 0.001. The fitness was also shown by the two measures of predictive power: Cox and Snell R Square = 0.38, and Nagelkerke R-Square = 0.508. After controlling for other variables in the model (Table 3), the odds of retention were significantly higher for patients at WHO clinical stage 1 compared to stage 4 (AOR, 1.325; 95% CI, 1.13–1.55). Compared to patients who received multiple months of ART, the odds of retention were much lower than for those who did not receive any (AOR, 0.01; CI, 0.00–0.01) and those who received a one-month supply (AOR, 0.22; CI, 0.20–0.23). Compared to those who had been on ART for 53 months or longer, the odds of retention were the highest for patients who had been on ART for less than 7 months (AOR, 7.52; CI, 6.64–8.52). Those on ART for 7 to <23 months had much lower retention (AOR, 2.43; CI, 2.23–2.65) and patients on ART for 23 to <53 months it was lower still (AOR, 1.36; CI, 1.25–1.47). The likelihood of retention was much higher for women who were not pregnant at the ART initiation (AOR, 2.80; CI, 2.04–3.85).

Compared to patients 55 years of age or older, the odds of retention were significantly lower for patients younger than 15 years (AOR, 0.35; CI, 0.30–0.42), and those aged 15 to <55 (AOR, 0.75; CI, 0.68–0.82). The odds of retention for females were twice those for males (AOR, 2.00; CI, 1.63–2.44). Both the geographic variables were also significantly associated with odds of retention, which were significantly higher for patients in Haut-Katanga province (AOR, 9.28; CI, 7.55 to 11.40), and significantly lower for patients in urban health zones (AOR, 0.75; CI, 0.59 to 0.94).

## 4. Discussion

In this study, the results showed an overall retention in care of 78.2%, indicating that the remaining 21.8% of PLHIV enrolled in the HIV programs were included in the patient attrition rate for the clinics. Inefficient retention and resulting disruptions to the continuum of care increase the risk of community transmission, and poor health outcomes including death and unsuppressed viral load [2,3,4,25]. Given that retaining PLHIV in care determines adherence to treatment and population-level prevention [6,26], our findings about retention rates should inform efforts of the HIV programs in DRC to fight against HIV/AIDS.

The higher odds of retention in care for patients at WHO clinical stage 1 and stage 2 compared to stage 4 are consistent with current research studies in other countries [1,26] and may be attributable to the high risk of death in patients at higher WHO clinical stages [26]. Aggressive testing and treatment along the model of universal test and treat (UTT) may be considered for reducing the disease progression [27]. Our findings about the lower odds of retention for patients who did not receive any ART or received a one-month supply compared to those who received multiple months’ supply were just as expected given that only those patients who receive a multiple-month supply were considered by the care providers as stable in treatment adherence and at the early stages of disease progression. These findings are similar to recent studies and are very encouraging for Sub-Saharan HIV programs that use community-based differentiated models that include multi-month ART dispensation to increase treatment adherence and lower patient attrition [28,29,30,31]. Previous research showed that treatment adherence is higher among patients with a lower CD4 count and adherence, in turn, helps patients manage their HIV better [32,33,34]. Reasons for failure to adhere to treatment are as diverse as the PLHIV themselves. The inability to make an autonomous decision regarding healthcare and dependence on adults for the administration of medications and transportation to treatment centers make children more vulnerable to failing to adhere to treatment [9].

Mancinelli and colleagues reported that educated, urbanized HIV-infected adults living far from program centers were at high risk of LTFU particularly if there were no maternal figure in the household [20]. Other studies showed the impact of distance from program centers [1,9], lack of education [16], CD4 cell count <350/mL [9,25], duration on ART of less than six months [25], advanced WHO clinical stage at initiation [35,36], being a female younger than 25 [37,38], and being male [13,25,35] on the ability of PLHIV to adhere to treatment and not become LTFU. We also found that the duration on ART had a negative association with the likelihood of retention, even after accounting for the WHO clinical stage of disease progression, and other covariates. Patients in the first quartile of duration on ART in the study population (<7 months) had over 7 times the odds of retention compared to those in the 4th quartile, i.e., those with 53 months or longer on ART.

Our study showed that pregnancy at the time of the start of ART was associated with lower odds of retention, which was consistent with some existing studies. For instance, a study of the association between pregnancy at enrollment into HIV care and loss to care among women in DRC showed that pregnant women were twice as likely to be lost to follow-up [13]. The greater risk of LTFU to women who were pregnant at the time of enrollment was attributable to competing demands on their financial resources, time, and attention for the management of pregnancy and HIV [39,40]. Reducing the LTFU in pregnant women should be a high priority for HIV prevention programs due to the risk of mother-to-child infection. Our study also showed that retention rates were higher for the clinics in Haut-Katanga province than in Kinshasa. However, this may have been due to a data limitation. There was a selection bias because the data available for Haut-Katanga were from health zones supported by a single implementing partner (SANRU), whereas the data for Kinshasa health zones were for ICAP, SANRU, and HPP.

Several strategies have been suggested to boost retention levels, including HIV prevention programs and policies that may use small financial rewards to advance telehealth and health informatics for better follow-up, contact tracing through appropriate apps on mobile phones [41], decentralized distribution of medications, multi-months dispensing, online counseling, and text reminders. Strategies currently used in HIV service programs to increase retention so that there is no disruption in care include the use of peer support groups such as ‘‘Community ART groups’’ and “Mentor Mothers” to provide psychosocial support to HIV-positive women, men, and children in their community [9]. Other types of opportunities within HIV programs to enhance retention is same-day ART diagnosis and treatment initiation. This strategy is useful because historically if patients received a positive test result and did not immediately begin treatment, they could easily become LTFU due to protocols that required a CD4 test before ART initiation. Upon recognizing this opportunity for LTFU, UNAIDS addressed it in the “90-90-90” guidelines by removing the need for a CD4 test before ART initiation. Starting ART at HIV diagnosis has a higher likelihood of retention in care relative to deferred therapy [16,18]. Additionally, HIV programs can increase retention by reinforcing community support organizations, which have helped retain ART patients in rural areas compared to other settings [9]. Capturing as much contact information as possible during the initial visit, updating patient contact information at every opportunity and reminding patients have been suggested in the literature for enhancing retention [11]. Initial visits may be opportunities to identify psychosocial issues and quick linkages to community support organizations [11]. The engagement of pregnant women before delivery may improve postpartum retention in care [42].

This study had some limitations. Like most quantitative studies, we lacked contextual information to appreciate fully the implication of our results. Second, in examining the factors associated with retention in care, we had a limited number of variables available in data designed primarily for recoding HIV services provided to the PLHIV. Therefore, not all potential confounders and predictors could be included in the analysis. Third, given that retention is affected by a host of socio-economic and lifestyle variables, our study could not take advantage of such factors as they were not available from the secondary data. Finally, the ideal variable for LTFU would have been person–month till outcome, but we had only the dichotomous variable LTFU, which by definition resulted in censored data. So we could not use survival analysis. However, since our findings were based on a large-scale robust dataset from HIV clinical service providers in two provinces, they are generalizable to HIV services throughout the DRC.

## 5. Conclusions

In this age of evidence-based public health, the results of this study may inform HIV care program planning and quality improvement efforts. The level of attrition in our study pointed to opportunities to improve the quality of service. Higher odds of retention for patients in WHO stages 1, 2, and 3 compared with those in stage 4 indicating AIDS, imply that HIV programs will benefit from aggressive testing and treatment to initiate timely intervention and control disease progression. This study highlighted other factors that influence retention and the potential reasons that explain the association of those factors with retention in care. We recognized that there are inherent limitations to the use of program data for examining the factors that influence retention rather than loss to follow-up. Therefore, we propose that additional data, preferably qualitative data collected from all concerned in the care process be included in future investigations.

## Figures and Tables

**Table 1:** Descriptive statistics for characteristics of clients in HIV/AIDS clinics of Haut-Katanga and Kinshasa Provinces, DRC, 2014–2019.

Patient Characteristics	Frequency	Percent
**Retention in care, as opposed to attrition**		
Attrition (LTFU/Died)	10,855	21.8
Remained in care	38,906	78.2
**Current Age**		
<15	3629	7.1
15 to <55	40,200	78.4
55 or older	7457	14.5
**Sex**		
Female	34,547	67.4
Male	16,739	32.6
**Pregnant at ART initiation**		
Not applicable/(Male or <15 years)	17,622	34.4
Not reported/Not available	16,500	32.2
No	15,644	30.5
Yes	1520	3.5
**WHO Clinical Stage**		
Unknown	6943	13.5
1	21,566	42.1
2	9595	18.7
3	11,565	22.6
4	1617	3.2
**Duration on ART in months**		
<7	11,914	23.2
7–21.99	13,301	25.9
22–52.99	13,222	25.8
53 or longer	12,849	25.1
**Whether multiple months of ART was received**		
None	1984	7.0
1	12,760	44.8
2 or more	13,718	48.2
**Rurality/Urbanicity**		
Urban	49,718	96.9
Rural	1568	3.1
**Province**		
Haut-Katanga	4660	9.1
Kinshasa	46,626	90.9

Abbreviations: WHO, World Health Organization; ART, antiretroviral therapy; LTFU, lost to follow up.

**Table 2:** Variation in retention for persons living with HIV by patients’ demographic and clinical characteristics.

Demographic and Clinical Characteristics	Retention in Care	Attrition (LTFU/Died)	*p-*Value
**Current Age**			**<0.001**
<15	73.2%	26.8%	
15 to <55	78.2%	21.8%	
55 or older	80.7%	19.3%	
**Sex**			**<0.001**
Female	77.4%	22.6%	
Male	79.9%	20.1%	
**Pregnant at ART initiation**			**<0.001**
Not applicable (Male or <15 years)	78.9%	21.1%	
Not reported/Not available	66.7%	33.3%	
No	88.6%	11.4%	
Yes	87.0%	13.0%	
**WHO Clinical Stage**			**<0.001**
Unknown	83.8%	16.2%	
1	79.7%	20.3%	
2	79.4%	20.6%	
3	72.4%	27.6%	
4	67.0%	33.0%	
**Duration on ART in months**			**<0.001**
<7	78.4%	21.6%	
7–21.99	84.5%	15.5%	
22–52.99	78.0%	22.0%	
53 or longer	71.7%	28.3%	
**Whether multiple months of ART was received**			**<0.001**
None	17.6%	82.4%	
One month	67.3%	32.7%	
Two or more months	84.8%	15.2%	
**Province**			**<0.001**
Haut-Katanga	93.7%	6.3%	
Kinshasa	76.6%	23.4%	
**Method of ART Initiation**			**<0.001**
Not Known	15.3%	84.7%	
New	81.0%	18.7%	

Abbreviations: WHO, World Health Organization; ART, antiretroviral therapy; LTFU, lost to follow up. NOTE: The *p*-values are for the Chi-square test; the bold *p*-values indicate a significance of differences at *p* < 0.05.

**Table 3:** Logistic Regression of Retention of Persons Living with HIV in Care (N = 51,286).

Demographic and Clinical Characteristics	AOR	95% CI for AOR		*p-*Value
**Current Age**				
<15	0.35	0.30	0.42	**<0.001**
15 to <55	0.75	0.68	0.82	**<0.001**
55 or older	—	—	—	
**Sex**				
Female	2.00	1.63	2.44	**<0.001**
Male	—	—	—	
**Pregnant at ART initiation**				
Not Applicable (Male or <15 years)	5.66	4.39	7.30	**<0.001**
Not reported/Not available	2.58	1.98	3.35	**<0.001**
No	2.80	2.04	3.85	**<0.001**
Yes	—	—	—	
**WHO Clinical Stage**				
Unknown	**0.67**	0.53	0.85	**<0.001**
1	**1.33**	1.13	1.15	**0.001**
2	**1.12**	0.95	1.32	**<0.001**
3	0.99	0.84	1.17	0.195
4	—	—	—	
**Duration on ART in months**				
<7	7.52	6.64	8.52	**<0.001**
7–21.99	2.43	2.23	2.65	**<0.001**
22–52.99	1.36	1.25	1.47	**<0.001**
53 or longer	—	—	—	
**Whether multiple months of ART was received**				
None	**0.01**	0.00	0.01	**<0.001**
One months	**0.22**	0.20	0.23	**<0.001**
Two or more months	—	—	—	—
**Rurality/Urbanicity**				
Urban	0.75	0.59	0.94	**0.012**
Rural	—	—	—	—
**Province**				
Haut-Katanga	9.28	7.55	11.40	**<0.001**
Kinshasa	—	—	—	—

Abbreviations: ART, antiretroviral treatment; AOR, adjusted odds ratios; CI, confidence interval. Notes: AORs in boldface indicate statistical significance at *p* < 0.05 when compared to the reference category, indicated by “—”.

## Data Availability

The program implementing partners required that data be destroyed after publication. Original data are archived with the original data owners mentioned in the methods section.
